# An Updated Review of the Molecular Mechanisms in Drug Hypersensitivity

**DOI:** 10.1155/2018/6431694

**Published:** 2018-02-13

**Authors:** Chun-Bing Chen, Riichiro Abe, Ren-You Pan, Chuang-Wei Wang, Shuen-Iu Hung, Yi-Giien Tsai, Wen-Hung Chung

**Affiliations:** ^1^Department of Dermatology, Drug Hypersensitivity Clinical and Research Center, Chang Gung Memorial Hospital, Taipei, Linkou, Keelung, Taiwan; ^2^Chang Gung Immunology Consortium, Chang Gung Memorial Hospital and Chang Gung University, Taoyuan, Taiwan; ^3^Whole-Genome Research Core Laboratory of Human Diseases, Chang Gung Memorial Hospital, Keelung, Taiwan; ^4^College of Medicine, Chang Gung University, Taoyuan, Taiwan; ^5^Graduate Institute of Clinical Medical Sciences, Chang Gung University, Taoyuan, Taiwan; ^6^Immune-Oncology Center of Excellence, Chang Gung Memorial Hospital, Linkou, Taiwan; ^7^Division of Dermatology, Niigata University Graduate School of Medical and Dental Sciences, Niigata, Japan; ^8^Department and Institute of Pharmacology, School of Medicine, Infection and Immunity Research Center, National Yang-Ming University, Taipei, Taiwan; ^9^Division of Pediatric Allergy and Immunology, Changhua Christian Hospital, Changhua City, Taiwan; ^10^School of Medicine, Kaohsiung Medical University, Kaohsiung, Taiwan; ^11^School of Medicine, Chung Shan Medical University, Taichung, Taiwan; ^12^Department of Dermatology, Xiamen Chang Gung Hospital, Xiamen, China

## Abstract

Drug hypersensitivity may manifest ranging from milder skin reactions (e.g., maculopapular exanthema and urticaria) to severe systemic reactions, such as anaphylaxis, drug reactions with eosinophilia and systemic symptoms (DRESS)/drug-induced hypersensitivity syndrome (DIHS), or Stevens–Johnson syndrome (SJS)/toxic epidermal necrolysis (TEN). Current pharmacogenomic studies have made important strides in the prevention of some drug hypersensitivity through the identification of relevant genetic variants, particularly for genes encoding drug-metabolizing enzymes and human leukocyte antigens (HLAs). The associations identified by these studies are usually drug, phenotype, and ethnic specific. The drug presentation models that explain how small drug antigens might interact with HLA and T cell receptor (TCR) molecules in drug hypersensitivity include the hapten theory, the p-i concept, the altered peptide repertoire model, and the altered TCR repertoire model. The broad spectrum of clinical manifestations of drug hypersensitivity involving different drugs, as well as the various pathomechanisms involved, makes the diagnosis and management of it more challenging. This review highlights recent advances in our understanding of the predisposing factors, immune mechanisms, pathogenesis, diagnostic tools, and therapeutic approaches for drug hypersensitivity.

## 1. Introduction

Drug hypersensitivity reactions are an important public health problem due to their potential to cause life-threatening anaphylaxis and rare severe cutaneous adverse reactions (SCAR). Drug hypersensitivity can be induced by immunologically mediated reactions (referred as drug allergies) as well as nonallergic direct mast cell-mediated drug reactions. Immunologic reactions have been divided into four categories according to the classical Gell and Coombs system: type I reactions, which are immediate in onset and mediated by IgE and mast cells and/or basophils; type II reactions, which are delayed in onset and caused by antibody- (usually IgG) mediated cell destruction; type III reactions, which are delayed in onset and caused by IgG drug immune complex deposition and complement activation; and type IV reactions, which are delayed in onset and are T cell mediated [1]. According to the World Allergy Organization (WAO), drug hypersensitivity reactions can also be categorized into immediate reactions and delayed reactions based upon the timing of the appearance of symptoms [2].

Immediate-type reactions usually occur within minutes or hours of drug exposure. The clinical manifestations range from pruritus, urticaria, angioedema, and bronchospasm to anaphylaxis. Type I reactions require the presence of drug-specific IgE or the portion of the drug that forms a hapten complex. Drug-specific IgE is produced upon the first exposure to the drug antigen, and then, it binds to basophils or mast cells with the high-affinity Fc receptor. Upon the next exposure to the same drug, two or more IgE molecules on the basophil or mast cell surface may then bind to one multivalent antigen molecule, initiating a series of cellular activation events. This activation causes the extracellular release of granules with preformed inflammatory mediators, including histamine, leukotrienes, prostaglandins, heparin, and other cytokines [3]. IgE-mediated immunologic drug allergy represents a smaller fraction of drug hypersensitivity compared with nonimmunologic drug hypersensitivity [4]. According to the WAO classification system, immunologic anaphylaxis can be caused by an IgE-mediated or non-IgE-mediated mechanism, whereas nonimmunologic anaphylaxis involves direct mast cell activation [2]. Regardless of the underlying mechanism, however, the clinical symptoms of both types of anaphylaxis are similar and often indistinguishable. The mechanism of immediate-type reactions is explained more fully later in this article. In this review, the terminology used to categorize “immediate” or “delayed” drug hypersensitivity is in accordance with the WAO classification system. At the same time, the immediate-type reactions discussed herein are composed of both IgE-mediated reactions as defined by the Gell and Coombs system, as well as non-IgE-mediated and nonimmunologic anaphylactic reactions.

Delayed-type reactions consist primarily of type IV reactions, which are T cell-mediated delayed-type drug hypersensitivity reactions. These reactions usually take several days or even weeks to manifest following drug exposure. These manifestations range from mild maculopapular exanthema (MPE), contact dermatitis, chronic allergic rhinitis, chronic asthma, nephritis, hepatitis, and fixed drug eruptions (FDEs) to life-threatening SCAR. SCAR includes drug reactions with eosinophilia and systemic symptoms (DRESS)/drug-induced hypersensitivity syndrome (DIHS), Stevens–Johnson syndrome (SJS) and toxic epidermal necrolysis (TEN), and acute generalized exanthematous pustulosis (AGEP) [5]. The MPE phenotype consists of self-limited diffuse erythematous macules and papules without systemic involvement [6]. DRESS syndrome, meanwhile, is characterized by cutaneous involvement with typical skin eruptions (e.g., exfoliative dermatitis and generalized maculopapular exanthema), fever, atypical lymphocytosis, eosinophilia, lymphadenopathy, and systemic involvement (e.g., liver involvement and kidney involvement). This hypersensitivity syndrome was first named after many different terms had already been used to describe the syndrome, with those terms, such as “anticonvulsant hypersensitivity syndrome,” “allopurinol hypersensitivity syndrome,” and “sulfone syndrome,” primarily depending on the culprit drug involved [7, 8]. The term “DRESS” was initially proposed by Bocquet et al. in 1996 in order to provide a more concise description of the syndrome and decrease the ambiguity resulting from the various terms previously used to refer to it [9]. That said, it should be noted that DRESS is also termed “DIHS” by Japanese experts, with the criteria of DRESS as defined by the RegiSCAR group and the criteria of DIHS as defined by Japanese experts being similar, except that HHV-6 reactivation is included in the diagnostic criteria for DIHS [10]. This nosology is somewhat confusing; however, there is a consensus that DRESS and DIHS are likely within the same disease spectrum. Specifically, patients with typical DIHS may represent a severe form of DRESS syndrome [11]. SJS and TEN (SJS/TEN) are characterized as a rapidly progressing blistering exanthema of purpuric macules and target-like lesions accompanied by mucosal involvement and skin detachment. SJS is defined as involving less than 10% body surface area skin detachment, SJS-TEN overlap as involving 10–29%, and TEN as involving more than 30% [12]. AGEP, meanwhile, typically presents as a sudden eruption of small nonfollicular pustules on a background of erythema with systemic involvement along with fever and neutrophilia [13].

Most forms of drug hypersensitivity involve T cell-mediated immune responses against specific drug/peptide antigens, leading to various clinical phenotypes. T cell receptor (TCR), CD4^+^, and CD8^+^ T cells are involved in the different delayed-type drug hypersensitivity reactions [14]. The molecular mechanisms and checkpoints for drug hypersensitivity include T cell activation and immune responses, cytotoxic proteins and cytokine/chemokine secretion, specific TCR clonotypes, impaired drug metabolism or clearance (e.g., the strong association of cytochrome P450 family 2 subfamily C member 9^∗^*3 (CYP2C9*^∗^*3)* with phenytoin-induced SCAR), and the cell death mechanisms (e.g., miR-18a-5p-induced apoptosis and annexin A1 and formyl peptide receptor 1-induced necroptosis in keratinocytes). In addition, genetic polymorphisms and specific *HLA* loci also play an important role (e.g., *HLA-B*^∗^*15:02* for carbamazepine- (CBZ-) induced SJS/TEN, *HLA-B*^∗^*58:01* for allopurinol-induced SCAR, and *HLA-B*^∗^*57:01* for abacavir-induced hypersensitivity reactions). Moreover, environmental factors, autoimmune disorders, and patients with a prior medical history of viral infection have also been reported to be implicated in susceptibility to drug hypersensitivity.

## 2. Clinical Perspectives and Variabilities in Severe Drug Hypersensitivity

### 2.1. Immediate-Type Hypersensitivity

Immediate-type hypersensitivity reactions may range from urticaria and angioedema to severe fatal reactions, such as bronchospasm and anaphylaxis. Anaphylaxis is a life-threatening systemic hypersensitivity reaction mainly mediated by mast cells and basophil activation via IgE-mediated, non-IgE-mediated, or nonimmunologic mechanisms. Drugs are the most common anaphylaxis triggers in adults, while foods are the most common triggers in children and teenagers [15]. The incidence of drug-induced anaphylaxis has been reported to range from 0.04 to 3.1%, with a mortality rate of around 0.65% [2]. NSAIDs are the main culprits, followed by beta-lactam antibiotics [16, 17]. Perioperative anaphylaxis also remains an issue due to the administration of various combinations of neuromuscular blocking agents (NMBAs), induction agents (e.g., propofol, etomidate, midazolam, and ketamine), and antibiotics [18, 19]. Nonsteroidal anti-inflammatory drugs (NSAIDs) (with the exception of pyrazolones) are believed to rarely be among the causes of IgE-mediated anaphylaxis, but such anaphylaxis is more commonly related to an aberrant arachidonic acid metabolism [20–22]. The non-IgE-mediated immunologic mechanisms can be mediated by IgG antibodies, as well as by complement or contact system activation, but non-IgE-mediated anaphylaxis is clinically indistinguishable from IgE-mediated anaphylaxis [23, 24]. The causes of non-IgE-mediated immunologic anaphylaxis include biologics, lipid incipients, and dextran [2]. In contrast, nonimmunologic anaphylaxis, previously regarded as a form of pseudoallergic drug reaction, involves the direct stimulation of mast cell degranulation. These reactions are limited to certain groups of drugs, including NSAIDs, such as aspirin, as well as opiates, vancomycin, quinolones, and NMBAs [24, 25]. For radiocontrast media-induced anaphylaxis, the mechanisms are not entirely clear and several mechanisms may be involved, including IgE-mediated or direct stimulating histamine release or the activation of the complement cascades [24, 26, 27].

Due to the complexity of NSAID-induced drug hypersensitivity, a panel of experts from the European Academy of Allergy and Clinical Immunology (EAACI) has proposed a classification and practical approach to cases of drug hypersensitivity caused by NSAIDs [28]. The most frequently occurring type of these cases is cross-reactive hypersensitivity, for which the mechanism is not immunological but, rather, is primarily linked to cycloxygenase-1 inhibition. This immunological type of NSAID-induced hypersensitivity includes NSAID-exacerbated respiratory disease (NERD), NSAID-exacerbated cutaneous disease (NECD), and NSAID-induced urticaria/angioedema (NIUA) [28]. NSAIDs can also induce immunological (noncross-reactive) hypersensitivity reactions, including IgE-mediated single-NSAID-induced urticaria/angioedema or anaphylaxis (SNIUAA), and T cell-mediated single-NSAID-induced delayed hypersensitivity reactions (SNIDHR). Both cross-reactive reactions and SNIUAA are immediate-type reactions [28].

### 2.2. Delayed-Type Hypersensitivity

#### 2.2.1. Drug Reactions with Eosinophilia and Systemic Symptoms (DRESS)/Drug-Induced Hypersensitivity Syndrome (DIHS)

There have been no large epidemiologic studies of DRESS/DIHS, a shortcoming which could be due to the fact that the term “hypersensitivity syndrome” was instead used before [5]. It could also be explained by the difficulty of diagnosing DRESS/DIHS, which presents with a complex natural course, a wide diversity of manifestations, and various laboratory abnormalities, and also because there is no specific code for this condition [29]. The incidence of anticonvulsant-related DRESS/DIHS is about one per 1000 to one per 10,000 new users [30]. DRESS/DIHS can occur in pediatric patients, but is more common in adults [31]. Antiepileptic agents and allopurinol are the most commonly reported offending medications [32]. The symptoms often begin 2 to 6 weeks after drug incubation [9]. Damage to multiple systemic organs may occur during the course of DRESS/DIHS syndrome. The liver is most commonly involved among the organs, with liver involvement having been found in 51–84% of patients [33, 34]. Renal involvement also occurs frequently, having been reported in 10–57% of patients [33, 34]. Lung involvement is the third most common type of systemic involvement and may present in various forms ranging from nonspecific symptoms to interstitial pneumonitis, pleuritis, and acute respiratory distress syndrome [35, 36]. Cardiac involvement, meanwhile, has been reported in 4–27% of patients with DRESS/DIHS [37]. This complication is likely associated with the fatal outcomes of the condition, especially when acute necrotizing eosinophilic myocarditis occurs [38]. Several other systemic organs can also be involved in DRESS/DIHS, including the gastrointestinal tract, pancreas, central nervous system, and thyroid, while multiple organ failure associated with disseminated intravascular coagulation or hemophagocytic syndrome may also occur [31, 39]. The overall mortality rate of DRESS/DIHS is around 10% [32]. The likelihood of mortality in cases of DRESS/DIHS is primarily determined by the degree of systemic involvement [35]. Tachycardia, leukocytosis, tachypnea, coagulopathy, gastrointestinal bleeding, and systemic inflammatory response syndrome (SIRS) have also been found to be associated with poor outcomes in DRESS/DIHS patients [33].

#### 2.2.2. Stevens-Johnson Syndrome (SJS)/Toxic Epidermal Necrolysis (TEN)

Large epidemiologic investigations of SCAR, especially SJS/TEN, have been performed in Europe beginning 30 years [40, 41]. The reported incidence rates of SJS/TEN for various countries and ethnicities have included 0.93–1.89 cases (Germany), 1.2 cases (France (TEN)), 1.4 cases (Italy), 5.76 cases (United Kingdom), 8.0 cases (Han Chinese), and 12.7 cases (United States) per million people per year [5, 40–45]. The large variation among these rates of incidence might be due to differences in the studies reporting them, including differences in the populations studied, generational differences, differing diagnostic criteria, and differing methodologies (such as the use of registration databases or electronic nationwide healthcare databases). SJS/TEN can occur in different age groups, but the incidences of SJS, SJS-TEN, and TEN appear to be lower in US children than in adults [46]. Racial disparities in SJS/TEN incidence were first reported by a large population-based study, which found that SJS/TEN is more strongly associated with people of nonwhite ethnicities, particularly Asians and blacks [42]. Pharmacogenetic studies, meanwhile, have pointed out that the strength of genetic associations is related to the prevalence with which susceptibility alleles are carried in different ethnic populations, such as *HLA-B*^∗^*15:02* and *HLA-B*^∗^*58:01* in Asians [47, 48]. Although the above classical examples partially explain the phenomenon of specific drug hypersensitivity in specific ethnicities with specific genetic factors, not all cases of drug hypersensitivity can be fully elucidated using this approach.

Cases of SJS/TEN are primarily induced by medications, but *Mycoplasma pneumonia* infection, viral infection, and collagen vascular diseases have also been found to account for a small portion of such cases [49–52]. The European ongoing case-control surveillance of the SCAR (EuroSCAR) group used a case-control study to identify the drugs carrying a high risk of such reactions and found that they included sulfonamides, aromatic convulsants, allopurinol, oxicam nonsteroidal anti-inflammatory drugs, and nevirapine [53]. Newly developed drugs, such as anticancer target therapies, also have the potential to induce SJS/TEN [54]. SJS/TEN induced by monoclonal antibodies targeting the coinhibitory immune checkpoint with antiprogrammed death-1 (PD-1) (nivolumab) and anticytotoxic T-lymphocyte-associated protein 4 (CTLA-4) (ipilimumab) has likewise been reported [55, 56]. Proton pump inhibitors, meanwhile, have been known to induce type I hypersensitivity reactions, but they carry some risk of inducing life-threatening type IV hypersensitivity reactions as well [57]. That risk, however, is mostly confined to the first 8 weeks drug exposure, after which the onset of SCAR is much less likely [53]. Meanwhile, the ALDEN (ALgorithm for Drug causality in Epidermal Necrolysis) has been used to provide structured assistance for the assessment of culprit drugs in SJS/TEN patients [58].

The mortality rates of the various forms of SJS/TEN are high, at approximately 10% for SJS, 30% for overlapping SJS/TEN, and 50% for TEN, for an overall rate of about 25% [34, 59]. Indeed, the mortality rate for cases of TEN has remained high, with reported rates of 15.8%–49.0%, even with the overall improvements to health care in recent decades [42, 44, 60]. A disease severity scoring system called SCORTEN (SCORe of Toxic Epidermal Necrolysis) built on seven independent variables (age > 40 years; presence of malignancy; body surface area involved > 10%; serum urea nitrogen level > 28 mg/dL; glucose level > 252 mg/dL; bicarbonate [HCO_3_] level < 20 mEq/L; and heart rate > 120 beats per minute) can be used to help predict mortality in individual cases of SJS/TEN [61, 62]. Modified versions of this scoring system may be needed for specific populations, like pediatric patients [63].

#### 2.2.3. Acute Generalized Exanthematous Pustulosis (AGEP)

The annual incidence of AGEP is estimated to be one to five per million [64]. The EuroSCAR group conducted a large case cohort study of 97 validated cases of AGEP [13]. The mean age of the patients was 56 years (range: 4–91 years) [13]. The list of drugs reported to have been involved is extensive, but certain medications such as aminopenicillins, pristinamycin, quinolones, terbinafine, diltiazem, antimalarials, and Chinese herbs are known to be associated with higher risks of AGEP [13, 65]. The mortality rate of AGEP has been reported to be about 4%, a relatively low rate compared to those of SJS/TEN and DRESS/DIHS [13].

## 3. Genetic Factors in Drug Hypersensitivity

### 3.1. Genetic Factors in Immediate-Type Drug Hypersensitivity

Genetic predisposing factors have been reported in cases of immediate-type drug hypersensitivity resulting from the use of beta-lactams, aspirin, and other NSAIDs. Interestingly, HLA class II genes (*HLA-DRA* and the *HLA-DRA|HLA-DRB5* interregion) have been linked to immediate reactions to beta-lactams (Table 1) [66]. The genetic variants of proinflammatory cytokines (*IL4*, *IL13*, *IL10*, *IL18*, *TNF*, and *IFNGR1*), the cytokine receptor (*IL4R*), the genes involved in the IgE/FceRI pathway (the galectin-3 gene (*LGALS3*)), and nucleotide-binding oligomerization domain (*NOD*) gene polymorphisms are also strongly associated with beta-lactam-induced immediate reactions (Table 2) [67–73].

The involvements of *HLA-DRA*, *ILR4*, *NOD2*, and *LGALS3* have also been further validated by a replication study [72]. *HLA-DRB1*^∗^*13:02* and *HLA-DRB1*^∗^*06:09* are associated, meanwhile, with aspirin-induced urticaria/angioedema [74]. In addition, *HLA-B44* and *HLA-Cw5* have also been reported to be associated with chronic idiopathic urticaria associated with aspirin- and/or NSAID-induced hypersensitivity [75]. Several genetic predisposing factors have been reported to be associated with immediate-type aspirin hypersensitivity, with those factors involving cytokines (TGFB1, TNF, and IL18) and the production and release of mediators (LTC4S, TBXA2R, PTGER4, FCER1A, MS4A2, FCER1G, and HNMT) [76, 77]. Immediate-type hypersensitivity to NSAIDs has also been reported to be associated with genes belonging to the arachidonic acid pathway (*ALOX5*, *ALOX5AP*, *ALOX15*, *TBXAS1*, *PTGDR*, and *CYSLTR1*) [72, 78]. However, the association of common genetic variations in histamine receptor genes was not found in patients with hypersensitivity to NSAIDs [79].

### 3.2. Genetic Factors in Delayed-Type Drug Hypersensitivity

Recently, the number of pharmacogenetic studies of HLA-associated drug hypersensitivity and related drug-induced syndromes, such as fixed drug reaction, delayed rash, lupus erythematosus, drug-induced liver disease, DRESS/DIHS, SJS, and TEN, has been increasing. These associations are usually drug and ethnic specific (Table 1), which implies that specific HLA molecules may have higher binding affinities for specific drug antigens and present the drug antigens to specific TCRs, causing a series of T cell activations and adverse immune responses.

#### 3.2.1. Aromatic Anticonvulsants

Aromatic anticonvulsants, such as carbamazepine (CBZ), phenytoin (PHT), oxcarbazepine (OXC), and lamotrigine (LTG), are known to carry higher risks of inducing SCAR. A strong genetic association between *HLA-B*^∗^*15:02* and CBZ-induced SJS/TEN was found in 2004 in Han Chinese (corrected *P* value = 3.1 × 10^−27^, odds ratio (OR) = 2504, and 95% confidence interval (CI) = 126–49,522) and has further been validated in cohorts of various other Asian populations including Thai, Indian, Malaysian, Vietnamese, Singaporean, and Hong Kongese cohorts [45, 80]. The *HLA-B*^∗^*15:02* allele has also been identified as the common risk factor for SJS/TEN caused by other aromatic antiepileptic drugs [81], such as PHT [82, 83], OXC [84], and LTG [85]. The association between *HLA* alleles and CBZ-induced SCAR is phenotype and ethnic specific. The *HLA-A*^∗^*31:01* allele is as specific predictor of CBZ-induced DRESS but not CBZ-induced SJS/TEN in Europeans and Han Chinese [86, 87]. In contrast, a strong association with *HLA-A*^∗^*31:01* was found in CBZ-induced cutaneous adverse drug reactions (cADR) but not only in DRESS/DIHS in Northern Europeans, Japanese, and Koreans [88–90]. In addition to HLA alleles, a genome-wide association study showed a strong association of CYP2C9^∗^3 with PHT-induced SCAR in patients from Taiwan, Japan, and Malaysia and this finding was further supported by evidence indicating the delayed clearance of plasma PHT levels in PHT-induced SCAR [91].

#### 3.2.2. Allopurinol

Allopurinol is a first-line drug used to treat gouty arthritis and urate nephropathy. In 2005, Hung et al. reported that *HLA-B*^∗^*58:01* was the genetic risk marker for allopurinol-induced hypersensitivity in Han Chinese (corrected *P* value = 4.7 × 10^−24^, OR = 580.3, and 95% CI = 34.4–9780.9) [92]. This correlation was subsequently validated among different populations, including various Asian and European populations [93–96]. The gene dosage effect of *HLA-B*^∗^*58:01* also influences the development of allopurinol-induced hypersensitivity (OR = 15.3 for *HLA-B*^∗^*58:01* heterozygotes and OR = 72.5 for homozygotes), and the strength of the *HLA-B*^∗^*58:01* association has been found to be correlated with the disease severity of allopurinol-induced hypersensitivity (OR = 8.5 for MPE, OR = 44.0 for SCAR) [97].

#### 3.2.3. Antiretroviral Drugs, Antibiotics, and Other Drugs

The antiretroviral drugs, such as abacavir and nevirapine, are also known to cause hypersensitivity reactions. The association with abacavir was first found in 2002 due to the significant association between the *HLA-B*^∗^*57:01* and abacavir-induced hypersensitivity reactions (corrected *P* value < 0.0001, OR = 117, and 95% CI = 29–481). The positive predictive value of *HLA-B*^∗^*57:01* for abacavir hypersensitivity reactions has been reported to be 55% in Caucasians [98, 99]. Nevirapine, meanwhile, has been associated with nevirapine-induced hypersensitivity or DRESS in patients with *HLA-DRB1*^∗^*01:01* in Western Australia [100], *HLA-B*^∗^*35:05* in Thailand [101], and *HLA-Cw8* in Japan [102]. In addition, several antibiotic-induced hypersensitivity reactions and pharmacogenomic associations have also been reported, such as sulfonamide-induced allergic reactions [103], penicillin-induced SCAR [104], *HLA-B*^∗^*13:01* and dapsone-induced hypersensitivity syndrome in Chinese [105], *HLA-B*^∗^*57:01* and flucloxacillin-induced liver injury [106], and *HLA-A*^∗^*02:01* and *HLA-DQB1*^∗^*06:02* and amoxicillin-clavulanate hepatitis [107]. Other pharmacogenomic associations include *HLA-B*^∗^*59:01* and methazolamide-induced SJS/TEN in Koreans and Japanese [108], *HLA-B*^∗^*73:01* and oxicam-induced SJS/TEN in Europeans [93], and *ABCB11*, *C-24T*, *UGT2B7*^∗^*2*, and *IL-4 C-590-A* and diclofenac-induced liver disease in Europeans [109, 110].

## 4. Cellular Immunology and Immune Mechanisms in Drug Hypersensitivity

### 4.1. Antigen Presentation and Processing

Drugs are considered to be foreign antigens and bind to the HLA/peptide/TCR complex to trigger immune and hypersensitivity reactions. There are four hypotheses regarding drug presentation mechanisms that have been proposed to explain how small drug antigens might interact with HLA and TCR in drug hypersensitivity: (1) the hapten theory, (2) the pharmacological interaction with immune receptors (p-i) concept, (3) the altered peptide repertoire model, and (4) the altered TCR repertoire model [111–115].

First, the hapten theory states that the culprit drugs or their reactive metabolites are too small to be immunogenic on their own, whereas they covalently bind to the endogenous peptides to form an antigenic hapten-carrier complex. The hapten-carrier complex is presented to the HLA molecule and then recognized by TCR, resulting in the induction of drug-specific cellular or humoral immune responses. The hapten theory has been shown to be valid in cases of penicillin-induced cADR [111, 116]. Second, the pharmacological interaction with immune receptor (p-i) concept postulates that drugs may directly, reversibly, and noncovalently bind to the HLA and/or TCR protein and bypass the classic antigen-processing pathway in antigen-presenting cells. Wei et al. previously found that CBZ/aromatic antiepileptic drugs can directly interact with *HLA-B*^∗^*15:02* protein. No intracellular antigen processing or drug metabolism was involved in the *HLA-B*^∗^*15:02* presentation of CBZ [112]. Oxypurinol, the reactive metabolite of allopurinol, provides another example of the p-i concept in that it can directly and immediately activate drug-specific T cells via the preferential use of *HLA-B*^∗^*58:01* without intracellular processing [113]. Third, the altered peptide repertoire model states that the culprit drugs occupy the position in the peptide-binding groove of the HLA protein, changing the binding cleft and the peptide specificity of HLA binding. Abacavir-induced hypersensitivity has been found to belong to this model, as the crystal structure of *HLA-B*^∗^*57:01* has been found to form complexes with abacavir and peptides [114, 115]. These studies showed that abacavir binds to the F-pocket of *HLA-B*^∗^*57:01* and alters the shape and chemistry of the antigen-binding cleft, thereby altering the repertoire of endogenous peptides and resulting in polyclonal T cell activation and autoimmune-like systemic reaction manifestations. Finally, the altered TCR repertoire model suggests that some drugs, such as sulfamethoxazole, directly interact with TCR, but not with the peptides or HLA molecules. The drug antigens bind to specific TCRs and alter the conformation of those TCRs, giving them the potential to bind to HLA-self peptide complexes to elicit immune reactions [117]. In this model, TCR is regarded as an initial drug interaction molecule, suggesting that TCR is as crucial as HLA molecules and contributes to the occurrence of drug hypersensitivity. Furthermore, viruses have also been proposed to participate in HLA/drug/TCR interactions, in that they may provide exogenous peptides for drug presentation and play important roles in cADR [116].

### 4.2. Cellular Immunology and Immune Molecules Involved in Drug Hypersensitivity

#### 4.2.1. Immediate-Type Drug Hypersensitivity

Immediate-type drug hypersensitivity can be mediated by IgE-mediated or non-IgE-mediated mechanisms [118]. IgE-mediated mechanisms are mediated by drug-specific IgE via an immune response to a hapten/carrier complex. In the primary drug sensitization, drug-specific IgE is formed when plasma cells are transformed from activated B cells and interact with T cells. In an allergic reaction, drug allergens bind to mast cells or basophils with high-affinity Fc receptors, to which drug-specific IgE is bound, causing degranulation of the mast cells or basophils that results in the release of various mediators, such as histamine, leukotrienes, prostaglandins, and cytokines [3]. Degranulation has recently been proposed to occur in two main forms that are related to reaction severity and progression: piecemeal degranulation and anaphylactic degranulation [2, 119]. Piecemeal degranulation is mediated through the upregulation of CD203c on basophils via the formation of small vesicles from the histamine-containing granules quickly shuttling to the plasma membrane to cause more severe and rapid reactions [120]. Anaphylactic degranulation results in the fusion of the main histamine-containing granules with the plasma membrane, releasing the entire contents of granules to the extracellular space and exposing CD63 on the surface of basophils [120].

The non-IgE-mediated immunologic mechanisms are mediated by IgG antibodies or by complement activation [23, 24]. IgG-mediated anaphylaxis has been established in mouse models, wherein the use of drugs with specific IgG bound to Fc*γ*RIII stimulates the release of platelet-activating factor (PAF) by basophils, macrophages, or neutrophils [24]. Although the IgG-mediated anaphylaxis mechanism has not been fully demonstrated in humans, some studies have shown that PAF is an essential mediator in such anaphylaxis [121]. In addition, a novel gain-of-function splice variant of Fc*γ*R Fc*γ*RIIA has been identified with the presence of IgG anti-IgA antibodies in patients with common variable immunodeficiency who developed anaphylaxis after intravenous immunoglobulin infusion [122]. Moreover, biological agents with IgA and infliximab have been shown to induce anaphylaxis in the absence of specific IgE but with high levels of specific IgG [123–125]. These observations also provide some additional evidence for IgG-mediated anaphylaxis. Furthermore, complement activation can be induced through the absence of agent-specific IgE or IgG antibody immunocomplexes [24]. This condition can be observed in patients undergoing hemodialysis with a new dialysis membrane, protamine neutralization of heparin, and polyethylene glycol infusion [23, 126]. Drugs solubilized in therapeutic liposomes and lipid-based excipients (such as Cremophor EL used as the diluent for older preparations of propofol and paclitaxel) can form large micelles with serum lipids and cholesterol to stimulate the complement system [23, 126]. This activation of complement mechanisms further causes the release of C3a, C5a, and C5b-9, which trigger, in turn, the activation of mast cells, basophils, and other cells via their specific receptors, resulting in degranulation and mediator release [24].

The nonimmunologic-type hypersensitivity reaction directly activates mast cell degranulation without involving the activation of the immune system. There are several specific agents that induce different mechanisms beyond the direct immunoglobulin-mediated activation or complement activation. Oversulfated chondroitin sulfate-contaminated heparin was found to have caused various cases of anaphylaxis around 2007-2008 via the direct activation of the kinin system with increased production of bradykinin, C3a, and C5a [127]. The triggering of factor XII-driven contact system activation-mediated bradykinin formation also plays a key role in anaphylaxis [24]. NSAIDs, including aspirin, can result in anaphylactic reactions via the inhibition of cyclooxygenase with a decrease in the production of prostaglandins and the increased generation of cysteinyl leukotrienes [23]. Vancomycin can directly activate mast cells and/or basophils, leading to the release of histamine [128]. This mechanism was suggested to be mediated via the calcium-dependent activation of phospholipase-C and phospholipase-A2 pathways [128]. Opiates (e.g., meperidine, codeine, and morphine) also cause histamine release via direct mast cell degranulation [129]. Recently, it was proposed that nonimmunologic hypersensitivity reactions may also be mediated through the MAS-related G protein-coupled receptor-X2 (MRGPRX2) in cases involving specific drugs, such as icatibant, neuromuscular blocking drugs, and quinolone antibiotics [25]. The interaction of certain drugs with this mast cell receptor can stimulate degranulation and the release of TNF-*α* and prostaglandin D2 (PGD2), among other molecules, leading to nonimmunologic anaphylactic reactions [25]. The mouse counterpart of MRGPRX2 that participates in peptidergic drug-induced pseudoallergic reactions has been newly identified and could potentially be applied in preclinical screening models [25, 130].

#### 4.2.2. Delayed-Type Drug Hypersensitivity

The main concept used to explain the pathomechanisms of delayed-type drug hypersensitivity consists of the view that specific T lymphocytes or natural killer (NK) cells are activated upon antigen recognition or Fas/FasL interaction and that various cytotoxic proteins, including perforin/granzyme B, and granulysin, are then released to attack keratinocytes or other cells, inducing skin rash or epidermal necrosis. In addition, several other cytokines/chemokines, including TNF-*α*, IFN-*γ*, GM-CSF, TARC/CCL17, IL-6, IL-8/CXCL8, IL-15, and IL-36, are also known to participate in the immune reactions of drug hypersensitivity. These cytokines/chemokines have been found to be highly expressed in the skin lesions, blister fluids, blister cells, peripheral blood mononuclear cells (PBMC), or plasma of patients. These immune mediators are responsible for the trafficking, proliferation, regulation, or activation of T lymphocytes and other leukocytes, thereby affecting the clinical presentations of drug hypersensitivity in various ways (Table 3).

*(1) Fas-FasL Interaction*. Fas ligand (FasL) belongs to the tumor necrosis factor (TNF) family. The binding of Fas and FasL plays an important role in regulating the immune system and is involved in the apoptosis of epidermal cells in patients with drug hypersensitivity. Briefly, upon Fas–FasL interaction, the Fas-associated death domain protein (FADD) is recruited and binds to the Fas–FasL complex. The FADD then recruits procaspase 8, bringing multiple copies of procaspase 8 together, which in turn autoactivate to become caspase 8, triggering the caspase cascade and resulting in intracellular DNA degradation [131]. Viard et al. proposed that a suicidal interaction between Fas and FasL, which are both expressed by keratinocytes, leads to the extensive necrosis of epidermal cells in individuals with SJS/TEN [132].

*(2) Perforin/Granzyme B*. A controversial hypothesis suggests that perforin and granzyme B play more important roles in the keratinocyte death in SJS/TEN than does the Fas–FasL interaction [133]. Granzymes are serine proteases that are released by cytoplasmic granules and can induce programmed cell death in the target cells. Upon activation, drug-specific cytotoxic T lymphocytes (CTL) and NK cells produce perforin, which can bind to and punch a channel through the cell membrane, promoting the entry of granzyme B into the target cells to activate the caspase cascade and the succeeding apoptosis [134]. Delayed reactions to drugs have shown that increasing levels of perforin and granzyme B are related to the disease severity of drug hypersensitivity [131].

*(3) Granulysin*. Granulysin is a cytolytic protein mainly released by CTL and NK cells. It functions to create holes in the cell membranes and thereby destroy target cells. In 2008, Chung et al. reported that 15 kDa secretory granulysin serves as a key mediator for the disseminated keratinocyte apoptosis seen in SJS/TEN [135]. In that study, the increased level of granulysin in blister fluids from the skin lesions of SJS/TEN patients was much higher than the levels of other cytotoxic proteins, such as perforin, granzyme B, and FasL, and depleting the granulysin reduced the cytotoxicity [135]. Further studies demonstrated that granulysin is strongly expressed in patients with drug-induced FDE, DRESS/DIHS, and SJS/TEN but not MPE [136–138].

*(4) TNF-α, IFN-γ, TARC, IL-15, and Other Cytokines/Chemokines in SJS/TEN, DRESS/DIHS, and AGEP*. TNF-*α* is a major proinflammatory cytokine and is produced by macrophages, T lymphocytes, NK cells, neutrophils, mast cells, and eosinophils. It regulates immune responses through the induction of cell apoptosis, activation, differentiation, and inflammation [139]. TNF-*α* was highly expressed and suggested to be responsible for the extensive necrosis of skin lesions of SCAR patients [140, 141]. IFN-*γ* is critical for both innate and adaptive immunity against viral and bacterial infection, and it is predominantly produced by CD4^+^ T helper cells, CD8^+^ CTL, and NK cells. IFN-*γ* was found to be increased in the skin tissue, blister cells, and plasma of patients with erythema multiforme, SJS, TEN, and DRESS/DIHS [131, 142, 143]. The immune mechanism of AGEP is not yet well understood. However, high levels of IL-8/CXCL8 production and the recruitment of neutrophils have been observed in the skin lesions of AGEP patients [144–146]. Mutations in the *IL36RN* gene encoding the IL-36 receptor antagonist (IL-36Ra) have also been identified in AGEP patients [147, 148]. DRESS/DIHS is characterized by leukocytosis with atypical lymphocytosis or eosinophilia [149]. Serum thymus and activation-regulated chemokine (TARC) was identified as a potential biomarker for early indication of the disease and a predictor of disease activity in DRESS/DIHS [150, 151]. Compared to patients with MPE and SJS/TEN, the TARC levels in patients with DRESS/DIHS are significantly higher during the acute phase and are correlated with skin eruptions [151]. Interleukin-15 (IL-15) is a cytokine that can induce the proliferation of NK cells and other leukocytes, and it has been found to be associated with the disease severity and mortality of SJS/TEN [138]. IL-15 has also been shown to enhance the cytotoxicity of cultured NK cells and blister cells from TEN patients [138]. In addition, other cytokines and chemokine receptors, including IL-2, IL-4, IL-5, IL-6, IL-8, IL-10, IL-12, IL-13, IL-18, CCR3, CXCR3, CXCR4, and CCR10, have been found to be upregulated in the skin lesions, blister fluids, PBMC, or plasma of drug hypersensitivity patients and to participate in the immune regulation of drug hypersensitivity [131, 138, 142, 143, 152–154].

*(5) Syndrome-Specific Effector Cells*. SJS/TEN is characterized by profound necrosis localized to the epidermis. Cytotoxic CD8 T cells, natural killer cells, and natural killer T cells producing the cytotoxic molecules, especially granulysin, which causes extensive keratinocyte death, are enriched in blister fluid samples from the skin lesions of patients with SJS/TEN. Granulysin serum levels are correlated with the severity of acute disease and mortality [135, 155]. These cytotoxic cells mediate the disease pathogenesis. It is shown that the function of regulatory T cells (Tregs) in SJS/TEN is inadequate, although present in normal frequency [156]. Immunological changes of DRESS/DIHS are characterized by the increase of atypical lymphocytes or eosinophils [149, 157]. Eosinophilia can be observed in 60–95% of DRESS/DIHS patients at the early stage of the illness [32, 157]. Most of DRESS patients had increased numbers of CD4^+^ T cells in the acute stage, which was associated with the severity of clinical symptoms, such as the extent of skin rash and reactivations of virus [158]. In addition, Tregs play important roles in DRESS/DIHS pathogenesis. Dramatic expansions of functional Tregs are found in the acute stage of DRESS/DIHS [156]. It is hypothesized that CD4^+^FoxP3^+^ T cells that are home to skin serve to limit the severity of acute disease by regulating the cytotoxic effector T cell responses. However, Treg responses eventually exhaust and this might contribute to ongoing viral replication and intermittent recurrence of clinical symptoms [156, 159]. In patients with AGEP, it is shown that the increased neutrophilic inflammatory processes are regulated by T lymphocytes, which is important in the pathogenesis. The recruitment of neutrophils was observed in the skin lesions of the patients with the late phase of disease development [144, 145].

## 5. Environmental Factors and Viral Infections in Drug Hypersensitivity

In addition to drug antigens, hypersensitivity reactions may be induced by other pathogens, such as *Mycoplasma pneumonia*, or viral infections. Virus-drug interactions associated with viral reactivation may also exist. For example, it is well known that human herpesvirus-6 (HHV-6) plays an important role in DRESS/DIHS. HHV-6 reactivation in patients with DRESS/DIHS may increase T cell activity after the initiation of the drug eruption and induce the synthesis of proinflammatory cytokines, including TNF-*α* and IL-6, which may in turn modulate the T cell-mediated responses [160]. Shiohara et al. reviewed the associations between viral infections and drug rashes, as well as the mechanisms by which viral infections induce drug rashes. The sequential reactivations of several herpes viruses (HHV-6, HHV-7, Epstein-Barr virus (EBV), and cytomegalovirus (CMV)) were found to be coincident with the clinical symptoms of drug hypersensitivity reactions [161]. Chung et al. reported that a new variant of coxsackievirus A6 (CVA6) acting as the causative agent may induce widespread mucocutaneous blistering reactions mimicking the features of erythema multiforme major or SCAR [52]. In addition, the virus may also provide exogenous peptides for drug presentation and participate in HLA/drug/TCR interactions. White et al. recently proposed that some patients may acquire primary infection via HHVs or other pathogens that in turn induce drug hypersensitivity [116]. The presence of HHV peptides in patients with high-risk HLA alleles may trigger the activation of cytotoxic T cells, thereby resulting in the development of SCAR. The pathogenic factors underlying the unusual presentations of drug hypersensitivity related to viral infections need to be further investigated.

## 6. Diagnostic Tools for Drug Hypersensitivity

### 6.1. Diagnostic Tools for Immediate-Type Drug Hypersensitivity

The most commonly used laboratory test for confirming a diagnosis of anaphylaxis consists of determining the patient's total serum tryptase level [162]. Serial measurements of tryptase levels can be taken during an anaphylactic episode, although measurements of the baseline level are considered to be most useful. In fact, while serial measurements of tryptase levels taken during an anaphylactic episode can serve as useful markers for evaluating these reactions, this approach is not used so widely in clinical practice due to the limitations involved in measuring tryptase during the acute phase of an episode. Elevated levels of histamine, the first mediator released by mast cells, in plasma or urine are also consistent with anaphylaxis [2]. However, plasma histamine levels are only transiently elevated, making them of little utility if the patient is evaluated more than 1 hour after onset of the episode [163]. At the same time, normal levels of tryptase or histamine do not preclude a diagnosis of drug hypersensitivity [15]. Other newly identified biomarkers, such as PAF and carboxypeptidase A3, bring hope for enhancing diagnostic accuracy, although their use remains experimental [15, 164].

For IgE-mediated hypersensitivity reactions, serum drug-specific IgE (sIgE) quantification and the basophil activation test (BAT) are frequently used to assess the culprit drug. The tests used to conduct sIgE immunoassays consist of radioallergosorbent testing (RAST), enzyme-linked immunosorbent assays (ELISAs), and fluoroenzyme immunoassays (FEIAs) [165]. While RAST or ELISAs are usually conducted using in-house techniques, FEIAs can be performed using commercial products, such as the ImmunoCAP-FEIA system [166–168]. Only a few products are available, meanwhile, for some drugs, particularly beta-lactam antibiotics [167, 169]. The sensitivity of the various immunoassays used has been found to average 62.9%, while the average specificity, PPV, and NPV are 89.2%, 83.3%, and 77.8%, respectively [168]. The average NPV is also relatively low in order to exclude allergic reactions and determine whether to perform a provocation test [170]. In comparison, the BAT test provides a higher average specificity (94.6%) and PPV (93.4%) than immunoassays [168]. The test uses flow cytometry after drug stimulation to determine the levels of basophil activation or degranulation markers; the upregulation of CD63 and CD203c is also usually measured [171]. Of note, the results of the BAT for aspirin/NSAID-induced hypersensitivity remain inconclusive due to the fact that they encompass both IgE-mediated allergic reactions and nonimmunological intolerances, limiting the use of the BAT in assessing non-IgE-mediated reactions [172]. Mediator release assays, meanwhile, measure the mediator released (histamine or leukotriene 4) in a supernatant upon cell activation after drug stimulation, but these assays have exhibited sensitivity and specificity levels too low for them to be recommended for the purposes of diagnosis [169, 173].

### 6.2. Diagnostic Tools for Delayed-Type Drug Hypersensitivity

The discovery of biomarkers for drug hypersensitivity is crucial for clinical purposes, including the early diagnosis and better prediction of this disease in order to prevent complications. We previously found granulysin to be a key cytotoxic molecule responsible for disseminated keratinocyte necrosis through the action of cytotoxic lymphocytes or NK-cell-mediated cytotoxicity with no direct cellular contact [135]. A significant correlation between the granulysin levels in blister fluids and clinical severity was also found [135]. In addition, the serum granulysin levels in patients with SJS/TEN have also been found to be significantly elevated before the development of skin detachment or mucosal lesions but then to drop rapidly within 5 days of disease onset [136]. As a potential marker for the early phase of SJS/TEN, a simple rapid immunochromatographic test for elevated serum granulysin was developed for immediate clinical use. Additionally, prolonged elevation of serum granulysin has also been found in DIHS patients, indicating that such elevation could possibly be used for the purposes of early diagnosis and predicting disease prognosis [174]. Furthermore, the levels of IL-15 were correlated with the disease progression and mortality of SJS/TEN at early stage [138]. Serum IL-15 levels can be further utilized as a marker for early diagnosis and prognosis monitoring [138]. For DRESS/DIHS, serum TARC levels in patients with DRESS/DIHS have been reported to be significantly higher than those in patients with SJS/TEN and MPE during the acute phase and to be correlated with skin eruptions [151]. TARC was thus identified as a potential biomarker for the early indication and disease activity of DRESS/DIHS and also for determining the prognosis of systemic severity of inflammation in drug eruptions other than SJS/TEN [150, 151]. For AGEP, meanwhile, no specific markers for diagnosing or predicting the disease have been identified at present [175].

Drug rechallenge is considered the gold standard for confirming a potential offending drug; however, its use is not practical due to the possible life-threatening consequences. As such, there is still no standard method for the confirmation of drug causality. Nonetheless, since HLA genotyping has been useful in screening for populations at risk for SCAR, HLA genotyping might be helpful for identifying culprit drugs via specific HLA alleles in at-risk populations [48, 176]. Several *in vitro* tests can be used to assist in the confirmation of drug causality, but the exact sensitivity and specificity of such tests are not well known [177, 178]. There are several tests currently available: the lymphocyte transformation test (LTT), ELISpot (Enzyme-linked immunospot assay) intracellular cytokine staining, and the enzyme-linked immunosorbent assay (ELISA) for the secretion of cytotoxic mediators including inflammatory cytokines, chemokine–chemokine receptors, IFN-*γ*, Fas–Fas ligand, perforin, granzyme B, and granulysin [179]. The LTT is a reproducible test for measuring the enhanced proliferative response of PBMC after the sensitization of T cells to a drug [180]. However, the sensitivity of the test has reportedly varied among various studies involving various drugs and clinical phenotypes and different timings for use of the test [181, 182]. The relevance of using the LTT in testing for SJS/TEN was relatively lower those using than DRESS/DIHS and AGEP [182]. Several modifications can help to increase the sensitivity of the LTT or ELISPOT, including stimulation with anti-CD-3/CD28 antibody-coated microbeads with IL-2, depletion of Treg/CD25hi cells, or the combined addition of anti-CTLA4 and anti-programmed cell death ligand 1 (PD-L1) antibodies to PBMC cultures [183–185]. IFN-*γ*-ELISpot showed a similar sensitivity (67%) and specificity in DRESS, but a higher sensitivity (71%) in SJS/TEN [179]. The data for an ELISA-based test used to detect granulysin showed better sensitivity (86%) in SJS/TEN, but the evidence was limited due to the small number of cases in the study [186]. Further larger studies will thus be needed to confirm both the sensitivity and specificity.

*In vivo* patch tests provide a low-risk method for reproducing delayed hypersensitivity with moderate reexposure of patients to suspected offending drugs [187]. The value of patch testing depends on the phenotypes and drugs involved. The sensitivity of such testing is generally <70%, but higher sensitivities have been reported for AGEP and for some selected populations such as abacavir-hypersensitivity, carbamazepine-induced SJS/DRESS, and fixed drug eruption patients [178, 187, 188]. The skin tests involving a prick or intradermal testing are considered to be crucial tools for evaluating drug hypersensitivity reactions, including IgE-mediated or delayed-type hypersensitivity, in both the European and American guidelines [22, 189–191]. However, these skin tests are usually not suggested for SCAR patients due to the risk of relapse, although late-reading intradermal tests are of value for AGEP patients and negative patch tests are of value for SCAR patients [187, 192].

## 7. Therapeutic Approaches in Drug Hypersensitivity

### 7.1. Therapeutic Approaches in Immediate-Type Drug Hypersensitivity

Anaphylaxis is a medical emergency and epinephrine is the treatment of choice for anaphylaxis to prevent its progression to a life-threatening condition [15, 193]. Epinephrine should be administered as soon as possible without delay to avoid mortality [194]. The intramuscular injection of epinephrine into the middle of the outer thigh is recommended to treat anaphylaxis in most settings and in patients of all ages [195]. Glucagon is indicated for patients receiving beta-blockers with refractory symptoms [196]. The use of corticosteroids was previously believed to decrease the risk of biphasic and protracted reactions; however, a systematic review of the literature failed to retrieve any randomized controlled trials to confirm their effectiveness [197]. An emergency department-based study also failed to find a decrease in the rates of return visits or biphasic reactions among patients treated with glucocorticoids [198]. These adjunctive therapies, including corticosteroids, antihistamines, and bronchodilators, could help to relieve symptoms, but should not be substituted for epinephrine or delay the use of epinephrine [199, 200].

### 7.2. Therapeutic Approaches in Delayed-Type Drug Hypersensitivity

For the treatment of severe delayed-type drug hypersensitivity, such as SJS/TEN, there are no optimal treatment guidelines. Thus far, in fact, only a few randomized trials that could be regarded as references to guide treatment have been conducted. The efficacy of systemic immunosuppressants or immunomodulatory treatments (e.g., corticosteroids, cyclosporine, intravenous immunoglobulins (IVIg), and plasmapheresis) still remains controversial. Systemic corticosteroids could be the most common treatment option, but the prior use of corticosteroids was found to prolong disease progression with no definite benefit in terms of survival [60, 201–203]. IVIg is one of the most commonly utilized therapies for SJS/TEN and is frequently the adjunctive therapy used for severe cases or pediatric patients [204]. In a meta-analysis, however, IVIg, even high doses of IVIg, failed to achieve statistically significant results supporting the conclusion that it is clinically beneficial [204, 205]. IVIg has been found to yield better outcomes in pediatric patients, but children with TEN usually have lower rates of mortality and better prognoses than adult patients [204, 206]. Cyclosporine, has been found to decrease the mortality rate and the progression of detachment in adults in an open-label phase II trial [207]. However, one recent cohort study revealed a statistically insignificant survival benefit for cyclosporine therapy compared to supportive care [208]. In contrast, the first meta-analysis of 7 studies regarding the effect on mortality of cyclosporine in the treatment of SJS/TEN showed a beneficial effect [209]. A trend identified in the same study also indicated that cyclosporine demonstrated better survival than IVIg [209]. There have also been an increasing number of case reports regarding the benefit of treatment with anti-TNF-*α* biologic agents for patients with TEN [210–215]. One recent systemic review showed that glucocorticosteroids and cyclosporine are the most promising therapies in terms of survival benefit, but no such benefits were observed for IVIg, plasmapheresis, thalidomide, cyclophosphamide, hemoperfusion, tumor necrosis factor inhibitors, or granulocyte colony-stimulating factor [216]. Meanwhile, IL-15 was demonstrated to be a major cytokine orchestrating SJS/TEN, indicating that further novel therapeutics including IL-15 blockers, the mammalian target of rapamycin (mTOR) inhibitors, and Janus kinase/signal transducers and activators of transcription (JAK/STAT) inhibitors hold promise for impacting various therapeutic targets [138, 217]. That said, further prospective, randomized controlled studies are needed to provide more definitive conclusions regarding treatment in patients with SJS/TEN.

Systemic corticosteroids have been considered the treatment of choice for patients with DRESS/DIHS, but they may be associated with an increased risk of complications such as opportunistic infections [218]. CMV and HHV-6 viral loads were also reported to be increased in patients receiving systemic corticosteroids, while EBV loads were higher in patients not receiving systemic corticosteroids [219]. Antiviral medications such as ganciclovir can be given in addition to steroids and/or IVIg in cases of severe disease with confirmation of viral reactivation [220]. Several previous studies have reported the effectiveness of treatment with IVIg [221]. However, the premature discontinuation of a prospective study regarding the role of IVIg treatment occurred due to severe adverse effects [222]. Plasmapheresis and other immunosuppressive drugs, such as cyclophosphamide, cyclosporine, interferons, muromonab-CD3, mycophenolate mofetil, and rituximab, may also be potential therapies [221]. Among the above treatments, the use of cyclosporine was successful in 2 recent cases with rapid response, and so, its use could be considered for patients with concerns about using longer courses of systemic corticosteroids [223]. Supportive treatment with topical steroid-based treatments for AGEP is suggested due to the mostly benign and self-limiting course of the condition [224, 225]. Meanwhile, the administration of systemic steroids for a short period can be considered for severe and refractory cases [175].

## Figures and Tables

**Table 1:** HLA association with various phenotypes of drug hypersensitivity in different populations.

Associated drug	HLA allele	Hypersensitivity reactions	Ethnicity	Reference
*Aromatic anticonvulsants*				
Carbamazepine	*B* ^∗^ *15:02*	SJS/TEN	Han Chinese, Thai, Indian, Malaysian, Vietnamese, Singaporean, Hong Kongese	[45, 82, 83, 226–230]
	*A* ^∗^ *31:01*	DRESS	Han Chinese, European, Spanish	[86, 87, 231]
	*A* ^∗^ *31:01*	DRESS/SJS/TEN	Northern European, Japanese, Korean	[88–90]
	*B* ^∗^ *15:11*	SJS/TEN	Han Chinese, Japanese, Korean	[89, 232, 233]
	*B* ^∗^ *59:01*	SJS/TEN	Japanese	[234]
	*B* ^∗^ *38:01*	SJS/TEN	Spanish	[231]
Oxcarbazepine Phenytoin	*B* ^∗^ *15:02*	SJS/TEN	Han Chinese, Thai	[81, 84]
	*B* ^∗^ *15:02*	SJS/TEN	Han Chinese, Thai	[81, 83]
	*B* ^∗^ *15:02*, *B*^∗^*13:01*, *B*^∗^*51:01*	SJS/TEN	Han Chinese, Japanese, Malaysian	[91]
	*A* ^∗^ *33:03*, *B*^∗^*38:02*, *B*^∗^*51:01*, *B*^∗^*56:02*, *B*^∗^*58:01*, *C*^∗^*14:02*	SJS/TEN	Thai	[235]
	*B* ^∗^ *51:01*	DRESS	Thai	[235]
	*B* ^∗^ *15:13*	DRESS/SJS/TEN	Malaysian	[236]
	*CYP2C9* ^∗^ *3*	DRESS/SJS/TEN	Han Chinese, Japanese, Malaysian	[91]
	*CYP2C9* ^∗^ *3*	SJS/TEN	Thai	[235]
Phenobarbital Lamotrigine	*B* ^∗^ *15:02*	SJS/TEN	Han Chinese	[81, 85, 237]
	*B* ^∗^ *38; B* ^∗^ *58:01*, *A*^∗^*68:01*, *Cw*^∗^*07:18*	SJS/TEN	European	[93, 238]
	*B* ^∗^ *38:01*	SJS/TEN	Spanish	[231]
	*A* ^∗^ *31:01*	SJS/TEN	Korean	[239]
	*A* ^∗^ *24:02*	DRESS/SJS/TEN	Spanish	[231]
*Allopurinol*	*B* ^∗^ *58:01*	DRESS/SJS/TEN	Han Chinese, Thai, Japanese, Korean, European	[92–96]
*Antiretroviral drugs*				
Abacavir	*B* ^∗^ *57:01*	HSS	European, African	[98, 99]
Nevirapine	*DRB1* ^∗^ *01:01*	DRESS	Australian	[240]
	*B* ^∗^ *35:05*	DRESS	Thai	[101]
	*B* ^∗^ *14:02*, *Cw*^∗^*08:01*, *Cw*^∗^*08:02*	HSS	Sardinian, Japanese	[102, 241]
	*C* ^∗^ *04:01*	DRESS/SJS/TEN	Malawian	[242]
*Antibiotics*				
Beta-lactam	*DR9*, *DR14.1*, *DR17*, *DR4*	Immediate-type drug hypersensitivity	Chinese	[243]
	*DRA* rs7192, *DRA* rs8084	Immediate-type drug hypersensitivity	Spanish, Italian	[66]
Cotrimoxazole	*B* ^∗^ *15:02*, *C*^∗^*06:02*, *C*^∗^*08:01*	SJS/TEN	Thai	[244]
Dapsone	*B* ^∗^ *13:01*	HSS	Han Chinese	[105]
Sulfamethoxazole	*B* ^∗^ *38:02*	SJS/TEN	European	[93]
Sulfonamide	*A* ^∗^ *29*, *B*^∗^*12*, *DR*^∗^*7*	TEN	European	[245]
*NSAIDs*				
Aspirin	*DRB1* ^∗^ *13:02*, *DRB1*^∗^*06:09*	Urticaria/angioedema	Korean	[74]
Aspirin and other NSAIDs	*DRB1* ^∗^ *11*	Urticaria/angioedema and hypotension/laryngeal edema	Spanish	[246]
Aspirin and other NSAIDs	*B* ^∗^ *44*, *Cw*^∗^*5*	Chronic idiopathic urticaria	Italian	[75]
Oxicam NSAIDs	*B* ^∗^ *73:01*	SJS/TEN	European	[93]
*Other drugs*				
Methazolamide	*B* ^∗^ *59:01*, *CW*^∗^*01:02*	SJS/TEN	Korean, Japanese	[108]

DRESS: drug reaction with eosinophilia and systemic symptoms; HSS: hypersensitivity syndrome; MPE: maculopapular exanthema; NSAIDs: nonsteroidal anti-inflammatory drugs; SJS: Stevens-Johnson syndrome; TEN: toxic epidermal necrolysis.

**Table 2:** Genetic association with pathogenetic pathways in immediate-type drug hypersensitivity.

Associated drug	Ethnicity	Cytokines/chemokines	Production and release of mediators	Drug metabolism	Others	Reference
*Beta-lactam antibiotics*	Korean	—	*MS4A2*	—	—	[247, 248]
	Chinese	*IL4R*, *IL4*, *IL10*, *IL13*, *IFNGR1*, *STAT6*	*—*	—	—	[69, 70, 249–252]
	Italian	*IL4R*, *IL13*, *NOD2*	*LGALS3*	—	—	[66, 68, 73]
	French	*IL4R*, *IL10*	*—*	—	—	[253]
	American	*IL4R*, *IL4*	*—*	*LACTB*	—	[67]
	Spanish	*IL4R*, *TNF*, *NOD2*	*LGALS3*	—	—	[66, 73, 254, 255]

*Aspirin*	Korean	*IL18*, *TGFB1*, *TNF*	*ALOX5*, *FCER1A*, *FCER1G*, *HNMT*, *TBXA2R*, *PTGER4*	—	—	[76, 256–263]
	Poles	—	*LTC4S*	—	*GSTM1*	[264]
	Venezuelan	—	*LTC4S*	—	—	[265]

*NSAIDs*	Spanish	—	*ALOX5*, *ALOX5AP*, *ALOX15*, *CTSLTR1*, *DAO*, *PPARG*, *PTGDR*, *TBXAS1*	—	*CEP68*	[78, 266, 267]
	French	—	*ALOX5*, *PTGER1*	—	—	[268]
	Brazilian	*IL4R*, *IL10*	*DAO*	—	*CTLA4*	[269]

**Table 3:** Delayed-type drug hypersensitivity-related cytokines and chemokines.

Phenotype	Cytokines/chemokines	Skin or blister	Plasma	PBMC	References
DRESS/DIHS	TNF-*α*		+		[160]
	IFN-*γ*	+	+	+	[270–272]
	IL-2			+	[270]
	IL-4			+	[270]
	IL-5			+	[270]
	IL-6		+		[160]
	IL-13			+	[270]
	IL-15		+		[138]
	TARC/CCL17		+		[273]

SJS/TEN	TNF-*α*	+	+	+	[131, 138, 141–143, 274, 275]
	IFN-*γ*	+		+	[131, 142, 143, 274]
	IL-2	+		+	[131, 143]
	IL-5	+			[143]
	IL-6	+	+	+	[143, 153, 154, 138]
	IL-8/CXCL8		+		[138]
	IL-10	+	+	+	[142, 153]
	IL-12	NS			[142]
	IL-13	+			[143]
	IL-15	NS	+		[142, 138]
	IL-18	+			[142]
	CCR3	+			[143]
	CXCR3	+			[143]
	CXCR4	NS			[143]
	CCR10			+	[152]

AGEP	IL-8/CXCL8	+			[145, 146]
	IL-36	+			[147, 148]
	GM-CSF			+	[145]

AGEP: acute generalized exanthematous pustulosis; CCR: C–C chemokine receptor; CXCR: CX chemokine receptor; DIHS: drug-induced hypersensitivity syndrome; DRESS: drug reactions with eosinophilia and systemic symptoms; IFN-*γ*: interferon-*γ*; IL: interleukin; NS: not significant; SJS/TEN: Stevens–Johnson syndrome and toxic epidermal necrolysis; TNF-*α*: tumor necrosis factor-*α*.
