# Erratum to “An Updated Review of the Molecular Mechanisms in Drug Hypersensitivity”

**DOI:** 10.1155/2019/2489429

**Published:** 2019-01-31

**Authors:** Chun-Bing Chen, Riichiro Abe, Ren-You Pan, Chuang-Wei Wang, Shuen-Iu Hung, Yi-Giien Tsai, Wen-Hung Chung

**Affiliations:** ^1^Department of Dermatology, Drug Hypersensitivity Clinical and Research Center, Chang Gung Memorial Hospital, Taipei, Linkou, Keelung, Taiwan; ^2^Chang Gung Immunology Consortium, Chang Gung Memorial Hospital and Chang Gung University, Taoyuan, Taiwan; ^3^Whole-Genome Research Core Laboratory of Human Diseases, Chang Gung Memorial Hospital, Keelung, Taiwan; ^4^College of Medicine, Chang Gung University, Taoyuan, Taiwan; ^5^Graduate Institute of Clinical Medical Sciences, Chang Gung University, Taoyuan, Taiwan; ^6^Immune-Oncology Center of Excellence, Chang Gung Memorial Hospital, Linkou, Taiwan; ^7^Division of Dermatology, Niigata University Graduate School of Medical and Dental Sciences, Niigata, Japan; ^8^Department and Institute of Pharmacology, School of Medicine, Infection and Immunity Research Center, National Yang-Ming University, Taipei, Taiwan; ^9^Division of Pediatric Allergy and Immunology, Changhua Christian Hospital, Changhua City, Taiwan; ^10^School of Medicine, Kaohsiung Medical University, Kaohsiung, Taiwan; ^11^School of Medicine, Chung Shan Medical University, Taichung, Taiwan; ^12^Department of Dermatology, Xiamen Chang Gung Hospital, Xiamen, China

In the article titled “An Updated Review of the Molecular Mechanisms in Drug Hypersensitivity” [1], the format of Table 1 was unclear. Additionally, “*B*^∗^*38:01* SJS/TEN Spanish [231]” should belong to Phenytoin not Carbamazepine. The updated table is shown below.

## Figures and Tables

**Table 1:** HLA association with various phenotypes of drug hypersensitivity in different populations.

Associated drug	HLA allele	Hypersensitivity reactions	Ethnicity	Reference
*Aromatic anticonvulsants*				
Carbamazepine	*B* ^∗^ *15:02*	SJS/TEN	Han Chinese, Thai, Indian, Malaysian, Vietnam, Singapore, Hong Kong	[45, 82, 83, 226–230]
	*A* ^∗^ *31:01*	DRESS	Han Chinese, European, Spanish	[86, 87, 231]
	*A* ^∗^ *31:01*	DRESS/SJS/TEN	Northern European, Japanese, Korean	[88–90]
	*B* ^∗^ *15:11*	SJS/TEN	Han Chinese, Japanese, Korean,	[89, 232, 233]
	*B* ^∗^ *59:01*	SJS/TEN	Japanese	[234]
Oxcarbazepine	*B* ^∗^ *15:02*	SJS/TEN	Han Chinese, Thai	[81, 84]
Phenytoin	*B* ^∗^ *15:02*	SJS/TEN	Han Chinese, Thai	[81, 83]
	*B* ^∗^ *15:02*, *B*^∗^*13:01*, *B*^∗^*51:01*	SJS/TEN	Han Chinese, Japanese, Malaysian	[91]
	*A* ^∗^ *33:03*, *B*^∗^*38:02*, *B*^∗^*51:01*, *B*^∗^*56:02*, *B*^∗^*58:01*, *C*^∗^*14:02*	SJS/TEN	Thai	[235]
	*B* ^∗^ *51:01*	DRESS	Thai	[235]
	*B* ^∗^ *15:13*	DRESS/SJS/TEN	Malaysian	[236]
	*CYP2C9* ^∗^ *3*	DRESS/SJS/TEN	Han Chinese, Japanese, Malaysian	[91]
	*CYP2C9* ^∗^ *3*	SJS/TEN	Thai	[235]
Lamotrigine	*B* ^∗^ *15:02*	SJS/TEN	Han Chinese	[81, 85, 237]
	*B* ^∗^ *38*, *B*^∗^*58:01*, *A*^∗^*68:01*, *Cw*^∗^*07:18*	SJS/TEN	European	[93, 238]
	*B* ^∗^ *38:01*	SJS/TEN	Spanish	[231]
	*A* ^∗^ *31:01*	SJS/TEN	Korean	[239]
	*A* ^∗^ *24:02*	DRESS	Spanish	[231]

*Allopurinol*	*B* ^∗^ *58:01*	DRESS	Han Chinese, Thai, Japanese, Korean, European	[92–96]

*Antiretroviral drugs*				
Abacavir	*B* ^∗^ *57:01*	HSS	European, African	[98, 99]
Nevirapine	*DRB1* ^∗^ *01:01*	DRESS	Australian	[240]
	*B* ^∗^ *35:05*	DRESS	Thai	[101]
	*B* ^∗^ *14:02*, *Cw*^∗^*08:01*, *Cw*^∗^*08:02*	HSS	Sardinian, Japanese	[102, 241]
	*C* ^∗^ *04:01*	DRESS/SJS/TEN	Malawian	[242]

*Antibiotics*				
Beta-Lactam	*DR9*, *DR14.1*, *DR17*, *DR4*	Immediate type drug hypersensitivity	Chinese	[243]
	*DRA* rs7192, *DRA* rs8084	Immediate type drug hypersensitivity	Spanish, Italian	[66]
Cotrimoxazole	*B* ^∗^ *15:02*, *C*^∗^*06:02*, *C*^∗^*08:01*	SJS/TEN	Thai	[244]
Dapsone	*B* ^∗^ *13:01*	HSS	Han Chinese	[105]
Sulfamethoxazole	*B* ^∗^ *38:02*	SJS/TEN	European	[93]
Sulfonamide	*A* ^∗^ *29*, *B*^∗^*12*, *DR*^∗^*7*	TEN	European	[245]

*NSAIDs*				
Aspirin	*DRB1* ^∗^ *13:02*, *DRB1*^∗^*06:09*	Urticaria/angioedema	Korean	[74]
Aspirin and other NSAIDs	*DRB1* ^∗^ *11*	Urticaria/angioedema and hypotension/laryngeal edema	Spanish	[246]
Aspirin and other NSAIDs	*B* ^∗^ *44*, *Cw*^∗^*5*	Chronic idiopathic urticaria	Italian	[75]
Oxicam NSAIDs	*B* ^∗^ *73:01*	SJS/TEN	European	[93]

*Other drugs*				
Methazolamide	*B* ^∗^ *59:01*, *CW*^∗^*01:02*	SJS/TEN	Korean, Japanese	[108]

DRESS: drug reaction with eosinophilia and systemic symptoms; HSS: hypersensitivity syndrome; MPE: maculopapular exanthema; NSAIDs: nonsteroidal anti-inflammatory drugs; SJS: Stevens-Johnson syndrome; TEN: toxic epidermal necrolysis.
